# Understanding the relationship between parenting style and chronic pain in adolescents: a structural equation modelling approach

**DOI:** 10.1186/s40359-021-00704-5

**Published:** 2021-12-24

**Authors:** Maryam Shaygan, Pardis Bostanian, Mina Zarmehr, Hamidreza Hassanipour, Maryam Mollaie

**Affiliations:** 1 Community Based Psychiatric Care Research Center, Faculty of Nursing and Midwifery, Shiraz University of Medical Sciences, P.O. Box 713451359, Shiraz, Iran; 2 Community Based Psychiatric Care Research Center, Shiraz University of Medical Sciences, Shiraz, Iran; 3 Student Research Committee, School of Medicine, Shiraz University of Medical Sciences, Shiraz, Iran

**Keywords:** Adolescents, Chronic pain, Emotional intelligence, Parenting style, Psychological distress, Self-esteem

## Abstract

**Background:**

Although the context of parenting has been incorporated into psychosocial pain research, very little attention has been paid to how parenting styles influence chronic pain in adolescents. The present study aimed to investigate the mediating role of self-esteem, emotional intelligence, and psychological distress in the association between parenting styles and chronic pain.

**Method:**

Seven hundred and thirty nine adolescents and their parents participated in this study. To identify adolescents with chronic pain, screening questions based on the 11th revision of the International Classification of Diseases were used. Baumrind parenting style questionnaire was used to assess the parenting style (permissive, authoritarian, and authoritative parenting styles). The structural equation modelling (SEM) was carried out in M-Plus version 6 to evaluate the direct, indirect, and total effects of different parenting styles on chronic pain.

**Results:**

The results in the SEM models revealed that only the indirect paths from authoritative and authoritarian parenting styles to pain through emotional intelligence (β_authoritative_ = − 0.003, 95% CI = − 0.008 to − 0.003; β_authoritarian_ = 0.001, 95% CI = 0.001 to 0.003) and psychological distress (β_authoritative_ = − 0.010, 95% CI = − 0.021 to − 0.004; β_authoritarian_ = 0.008, 95% CI = 0.004 to 0.016) were significant. Indirect paths from permissive style to pain and the mediating role of self-esteem were not significant.

**Discussion:**

Emotional intelligence and psychological distress significantly mediated the effects of authoritative and authoritarian parenting styles on chronic pain. The current results support the notion that interventions targeting effective parent–adolescent communication may be an important part of chronic pain management in adolescents. Moreover, the results provide rationale for targeting emotional intelligence and psychological distress in adolescents by explicitly teaching effective communication skills, expressing opinions and minds, and emotion regulation strategies.

## Background

Pediatric chronic pain is prevalent, disabling, and costly [1, 2]. It is pain that persists or recurs for longer than 3 months and is associated with significant emotional distress and functional disability [3]. In a recent study among adolescents across 42 countries, on average 20.6% of adolescents reported weekly chronic pain in at least two sites [1]. Continuous experience of pain in adolescents is associated with serious negative impacts such as sleep problems, poor school performance, low self-esteem, and disturbed social activities [2, 4, 5].

According to the biopsychosocial model, chronic pain is never a mere sensory perception but is a complex biopsychosocial condition that is affected by a wide range of psychosocial factors [6]. Therefore, we should think of those factors which are associated with chronic pain and the way they influence pain development in adolescents to effectively prevent the development of chronic pain in this population.

There is substantial evidence supporting the impact of parent emotions, cognitions, and behaviors on pediatric chronic pain [7, 8]. A rich body of research has identified family conflict [8], reinforcement of pain behaviors [8], parental pain catastrophizing [9], parental distress [10], and parental bonding style [11] as possible factors associated with adolescents' experience of chronic pain. Moreover, considerable empirical research supports that exposure to a dysfunctional parenting style during childhood increases the future risk of various forms of psychosomatic disorders [12–14]. However, there have been still few studies addressing the relationship between parenting style and chronic pain [5, 15]. Parenting style is defined as a set of parental behaviors that influences the development of their children [16]. Baumrind has identified three initial parenting styles including authoritative, authoritarian, and permissive [17, 18]. Authoritative parenting style is characterized by high scores in both demandingness and responsiveness, authoritarian parenting style by high scores in demandingness and low in responsiveness, and permissive parenting style by high scores in responsiveness and low in demandingness [17]. Authoritative parents are considerably more successful in protecting their children from behavior problems and in generating competence [17, 18]. Conversely, dysfunctional parenting behaviors associated with authoritarian and/or permissive parenting are associated with negative impacts on child development from infancy through adolescence [17, 19].

A small but growing body of research suggests that dysfunctional parenting styles, especially those characterized by low care and high overprotection (authoritarian parenting style), may influence development or maintenance of chronic pain in adolescents [5, 15, 20]. In their recent study, Shibata et al. found that parental excessive overprotection (affectionless control) during childhood was 2–3 times higher in the chronic pain patients than in the group of community-dwelling subjects without chronic pain, after controlling the demographic variables [15]. Anno et al. also suggested that a high level of parental overprotection was associated with an increased risk of chronic pain [11]. One other study demonstrated that authoritarian parenting was positively associated with adolescent’s chronic pain, and authoritative parenting was negatively associated with adolescent’s chronic pain. It was reported that the mean score of authoritative parenting among healthy adolescents was higher than those with the chronic pain. Inversely, the mean score of authoritarian parenting in adolescents with chronic pain was higher than their healthy peers [5]. However, the impact of parenting styles on health problems varies by cultural and social contexts in which it occurs [21]. For example, permissive parenting has been suggested to be tolerable to some extent in some cultural contexts [21]. It was reported that health variables in adolescents are more associated with authoritative and authoritarian than with permissive parenting style [22, 23]. However, further research on adolescents from different cultures is needed to investigate the possible impact of different parenting styles on pediatric chronic pain.

Apart from the established direct relations between parenting styles and chronic pain, the underlying mechanisms in these associations are not yet entirely clear [8, 9]. Some theoretical models have been developed to describe associations between family functioning and pediatric chronic pain. According to Palermo and Chambers’ model, individual factors such as adolescents’ emotional functioning may mediate the associations between family functioning and pediatric pain [24].

One of the emotional strengths that has been proposed as an important element in the processing of emotional information during the subjective experience of pain [25] is emotional intelligence. Emotional intelligence (EI) is defined as the ability to monitor and manage one’s own and others’ feelings and emotions to be able to communicate with others effectively [26]. Research has shown that individuals who accurately manage their emotions report fewer physical symptoms and less illness [27, 28]. There is some evidence that individuals with high EI have greater self-efficacy in managing their pain [25]. Some research has also proposed that EI might facilitate health outcomes through the use of different adaptive coping strategies and the ability to manage negative affect against stress [25, 29]. It is well-established that EI is considerably affected by the parenting styles [30]. In a later study, Nguyen and colleagues have shown that over-protectiveness or authoritarianism from mothers during childhood is related to lower EI, while the warmth and care of parents are related to higher EI among adolescents [30].

Another psychological concept in explanation of the association between parenting style and chronic pain might be the psychological distress. There is increasing evidence that parenting style affects psychological distress, characterized by symptoms of depression and anxiety, in children and adolescents [21, 31]. Based on Baumrind’s theory [32], children who have authoritarian parents have a tendency to be unhappy and vulnerable to stress. Various meta-analyses and reviews have also concluded that authoritarian, and in part, permissive parenting styles (both known as dysfunctional parenting styles) are associated with the presence of depression and anxiety in children and adolescents [21, 31, 33]. Inversely, parental warmth and authoritative parenting have been associated with less psychological distress and, in general, better psychological adjustment among this population [21, 31]. Psychological distress is, in turn, associated with persistent pain and likely predisposes an adolescent to it [34]. It has been well-established that negative emotions may trigger, maintain, or exacerbate pain [35]. Studies indicate that individuals with high negative affect have a heightened perception of clinical pain [36–38] and experimentally induced pain [25]. For the relationship between depression and pain, several mechanisms such as shared genetic vulnerability, neurobiological processes, and environmental factors have been proposed [39, 40]. Anxiety may also exert pain-inducing effects by mechanisms such as increasing awareness and altered perception of physical sensations [41].

Self-esteem which is defined as a person's overall evaluation of self [42] was also found to be associated with chronic pain [7]. Resilience-risk model for pediatric chronic pain has highlighted self-esteem as a resilience resource that may promote pain-related coping, pain management, and adjustment [7]. According to this model, self-esteem facilitates problem-solving, pain-related self-efficacy, sense of controllability, and active coping with pain [7]. It has been suggested that adolescents with high self-esteem are more likely to be involved in close relationships, which can increase emotional support as well as confidence regarding managing their illness [43]. One of the factors that shape the development of self-esteem among adolescents is parenting style. Authoritative parenting style has been suggested as the best parenting style which considerably has a positive impact on self-esteem of children. Conversely, authoritarian parenting style has a negative effect on the self-esteem [44].

## Current study

Although a growing body of research supports the association between parenting styles and health variables, little attention has been paid to how parenting styles influence chronic pain in adolescents [8]. To our knowledge, there has been no published original research to investigate how chronic pain is influenced by the manner in which individuals are raised by their parents. Hence, research is needed to better understand the exact mechanisms by which parenting styles exert their influence on adolescents’ pain.

The current study aims to explore innovative models linking parenting styles to chronic pain in adolescents. Specifically, it explores the mediating role of emotional intelligence, psychological distress, and self-esteem in the association between parenting styles and chronic pain. The present study used a powerful statistical tool (i.e., structural equation modelling) to examine multiple relationships among studied variables. Structural equation modelling provided a unique opportunity to investigate multiple hypotheses while simultaneously controlling for error. We hypothesized that emotional intelligence, psychological distress, and self-esteem would mediate the relationship between parenting style and chronic pain in adolescents. It was assumed that the proposed associations are significant for authoritative and authoritarian parenting styles. Exploring the direct and indirect effects of parenting styles on chronic pain might be an important step in providing more insight into questions relevant to the field of pediatric chronic pain.

## Method

### Participants and sampling

A cross-sectional, observational study was conducted on adolescents (12–19 years old) in Shiraz, Iran. Nine hundred and twenty adolescents were randomly selected to participate in this study. A multistage clustering sampling method was performed. First, two primary and two secondary schools were randomly selected (using simple random sampling) from each of the 4 delivery areas in Shiraz. At each school, 9–10 students were randomly selected (using systematic random sampling) at each grade. Totally, 920 adolescents were randomly selected from all 16 schools identified. The inclusion criteria for adolescents had an age range of 12–19 years, studied at one of the primary or secondary schools in Shiraz and had the ability to complete questionnaires. The following exclusion criteria were set: having no contact with any of the parents, having chronic physical illness not related to pain (e.g. asthma), and the presence of mental disorders or developmental disorders in adolescents based on their parents’ reports.

All adolescents and their parents who willingly participated in this study were informed about the research project and optional withdrawal from the study, and they were provided with written informed consent. The adolescents’ questionnaires were completed in the classroom during school hours in the presence of the researcher(s). The parents’ form/questionnaire (Baumrind parenting style questionnaire and consent form) were filled in by the most knowledgeable parent at home and returned to the teachers/schools. The data were collected anonymously and parents’ and adolescents’ questionnaires paired by number. We attempted to increase enthusiasm for participating in this study by explaining its aims. Ethical approval was obtained from the Ethics Committee of the Shiraz University of Medical Sciences (IR.SUMS.REC.1396.S795).

### Measures

In addition to the standard socio-demographic assessment (age, sex, parents’ job and educational level), the following variables were assessed.

#### Chronic pain assessment

To identify adolescents with chronic pain, we used three screening questions based on the 11th revision of the International Classification of Diseases (ICD‐11) [3, 20]: (a) “Are you currently troubled by pain or discomfort, either all the time or on and off?” (b) Have you had this pain or discomfort for more than 3 months?” (c) “Does it affect your life and activities?”. Additional information about the pain was requested if adolescents experienced pain lasting for at least 3 months: (d) any common causes of pain (if they had been diagnosed with headache, rheumatoid arthritis, back problems, abdomen problems, injury, etc.), (e) frequency of pain (answer categories: permanent, one or more attack per day, one or more attack per week, one or more attack per month), and (f) pain history (the number of months since they experienced pain). The average intensity of pain during the last 2 weeks was assessed with a numerical rating scale on a scale of 0–10, with 0 being no pain and 10 being the worst pain possible. In our previous study, the quantitative face validity of each item was confirmed by calculating the item impact score [4, 5]. The impact scores showed that all the questions had a score equal to or greater than 1.5. The content validity of the assessment tool was also approved qualitatively and quantitatively. The test–retest reliability was confirmed by calculating the correlation coefficients for each item. All questions exhibited correlation coefficients ≥ 0.74.

#### Emotional intelligence

Emotional intelligence (EI) was measured by the Persian version of the Trait Emotional Intelligence Questionnaire–Adolescent Short Form (TEIQue–ASF) [26]. This questionnaire is a scientific measurement instrument providing a comprehensive assessment of emotional intelligence. This self-report inventory contains 30 short statements, two for each of the 15 traits EI facets [45]. Participants were asked to respond to the degree of agreement to each item on a scale of 1 (Completely disagree) to 7 (Completely agree). TEIQue–ASF scores can range from 30 to 210. Higher scores on TEIQue–ASF indicate higher levels of trait emotional intelligence [26]. The Persian version of TEIQue–ASF has been found to have acceptable internal consistency (Cronbach’s alpha = 0.86) [46]. Convergent/Divergent validity of the Persian version of TEIQue–ASF was confirmed by a significant positive correlation between TEIQue–ASF and extraversion and a negative correlation with neuroticism and psychoticism, ranging from 0.1 to 0.5 (*P* < 0.05) [46]. This questionnaire was found to have a good internal consistency (coefficient omega = 0.86) in the present study.

#### Self-esteem

Self-esteem assessment was done by using Rosenberg’s Self-Esteem Scale (RSES) [42]. It is a brief 10- item questionnaire with items scored on a four-point Likert scale from 1 “Strongly disagree” to 4 “Strongly disagree”. The total score ranges from 10 to 40 with higher scores indicating higher self-esteem [42]. Cronbach’s alpha of the Persian version of RSES was 0.84 [47]; thus, it is reliable to use for Iranian population. The construct validity of the Persian version of RSES was confirmed by correlating its total score with Death Obsession Scale (r = − 0.34) [47]. Factor analysis of RSES scores confirmed the unidimensionality of the scale [48]. This scale has an adequate internal consistency (coefficient omega = 0.85) in the present sample.

#### Psychological distress

Psychological distress was assessed by the 21-item Depression, Anxiety, and Stress Scale (DASS-21) [49]. It consists of three 7-item subscales (depression, anxiety, and stress) using a 4-point Likert scale ranging from 0 (Never) to 3 (Almost always). The total score of all 21 items ranges from 0 to 63. Higher scores represent more psychological distress. The Persian version of the scale demonstrated good internal consistency (Cronbach’s alpha = 0.94) [50]. In a study by Asghari et al. (2008), a 3-factor model for the Persian version of DASS-21 was supported, and convergent validity and discriminant validity of the three subscales of the Persian version of DASS-21 were confirmed [32]. This scale demonstrates an excellent internal consistency in the present study (coefficient omega = 0.91).

#### Baumrind parenting style questionnaire

Baumrind parenting style questionnaire was used to assess the parenting style [32]. It consists of 30 items (each of permissive, authoritarian, and authoritative parenting styles contains 10 items). In this questionnaire, the parents’ opinions are measured on a 5-degree Likert scale and three separate scores are achieved by summing the scores of the questions of each style [32]. In a study by Farahini et al. (2014), the following Cronbach coefficient alpha values were obtained for each of the styles: 0.76 for permissive style, 0.72 for authoritarian style, and 0. 74 for authoritative style [51]. In a study by Minaei and Nikzad (2017), the validity of the Persian version of Baumrind parenting style questionnaire was confirmed [52]. They showed that all items of the Persian version of this questionnaire were loaded significantly on three factors and explained 30.47% of the variance in the measure. In the present study, the following coefficient omega values were obtained for each of the styles: 0.70 for permissive style, 0.73 for authoritarian style, and 0. 80 for authoritative style.

### Statistical analyses

Descriptive statistics such as means and SDs for continuous variables and frequencies and percentages for categorical variables were used for socio-demographic characteristics of the adolescents.

Before conducting structural equation modelling approach, data were checked for outlier values and normality assumption. Outliers were identified using a Z score cutoff of 3.29 as proposed by Field (2013) [53]. In order to test for normality of the distribution of each variable, Skewness and Kurtosis tests were applied. An absolute skew value larger than 2 or smaller than 2, or an absolute kurtosis value larger than 7 or smaller than 7 were used as reference values for determining substantial non-normality [54]. Bivariate correlations between all the measures were calculated in order to test the assumption of multicollinearity [55]. It is assumed that highly correlated variables (r > 0.90) indicate multicollinearity [56]. Bivariate correlations were also used to assess the potential relationship of any measure with socio-demographic (age, sex, etc.) variables. Socio-demographic variables showing a significant relationship with any of the measures were considered as a potential covariate in the analyses. SPSS version 22 (SPSS, Inc., Chicago IL, USA) was used to compute these analyses.

We applied the structural equation modelling (SEM) to evaluate the direct, indirect and total effects of each independent variable (permissive, authoritarian, and authoritative parenting styles) on the dependent variable (chronic pain). Three separate SEM models were performed to estimate the effects of each independent variable on pain in the presence of three mediating variables (self-esteem, emotional intelligence, and psychological distress). Independent variables and mediators were analyzed as continuous variables. Chronic pain was examined as a binary outcome variable (Yes/No). The three hypothesized models were tested by means of path analysis. Missing data were managed by multiple imputation using Bayesian analysis [57, 58]. There were a total of 196 (26%) missing data. In total, 5 imputed datasets were used.

Path models were evaluated with Bayesian estimator. Bayesian approach constructs credible intervals of indirect effects for simple as well as complex mediation models, and does not require the assumption of normality in the sampling distribution of estimates [59]. Path coefficients were significant when the Bayesian 95% credibility interval did not include zero [60]. Bayesian estimates of all the parameters were obtained after 10,000 MCMC (Markov Chain Monte Carlo) iteration runs with 2000 iteration burn-in [58, 60]. The non-informative prior distribution was assigned because there was no knowledge about given parameters [61]. Posterior Predictive *P*-value (PPP) method was applied to determine the hypothesized model’s fit. An excellent model fit is determined by a PPP value close to 0.50, and zero (0) falling near the middle of the 95% confidence interval [62, 63]. SEM models were carried out in M-Plus version 6.

## Results

One hundred and eighty-one of the 920 adolescents who were randomly selected to participate in the study dropped out: 61 adolescents because they did not return the self-report instruments or consent forms, and 120 adolescents because of ineligibility or fulfilling exclusion criteria.

A total of 739 adolescents were included in this study. The mean age of the adolescents was 14.98 ± 1.37 years, and about two-thirds were girls (62.7%). The majority of mothers (45%) and fathers (37.8%) had high school diploma. The highest percentage of mothers and fathers were unemployed (84.2%) and employed (83%), respectively (Table 1).

**Table 1:** Sample characteristics (n = 739)

Variables	Value n (%)
Sex	
Female	463 (62.7%)
Mother's education	
High school or less	271 (36.7%)
Diploma	333 (45.1%)
University	135 (18.3%)
Father's education	
High school or less	253 (34.2%)
Diploma	279 (37.8%)
University	207 (28%)
Mother's job	
Working	117 (15.8%)
Non-working	622 (84.2%)
Father's job	
Worker/employer	613 (82.9%)
Retired	100 (13.5%)
Unemployed	26 (3.5%)

No outlier values on the variables were found and the normality assumption was not established. The results of collinearity statistics showed that correlations coefficients of all variables with each other ranged from 0.01 to 0.64, indicating that multicollinearity was not present (Table 2). Age, mother's education, and father's education were significantly associated with self-esteem, emotional intelligence, and authoritarian parenting style (*P*-value < 0.05, Table 2). Age was also significantly associated with psychological distress and permissive parenting style (*P*-value < 0.05, Table 2). Therefore, age, mother's education, and father's education were considered as covariates in the mediation analyses. There were no significant relationships between other socio-demographic variables and pain, parenting styles, and psychological variables (Table 2).

**Table 2:** Bivariate correlations between study variables

Variables	(1)	(2)	(3)	(4)	(5)	(6)	(7)	(8)	(9)	(10)	(11)	(12)	(13)
(1) Chronic pain	1	− 0.19**	− 0.26**	0.30**	− 0.11**	0.10**	0.005	0.04	0.04	− 0.06	− 0.06	0.004	− 0.02
(2) Self-esteem		1	0.60**	− 0.63**	0.22**	− 0.15**	− 0.01	− 0.13**	0.008	0.11**	0.14*	0.01	− 0.03
(3) Emotional intelligence			1	− 0.64**	0.20**	− 0.10*	− 0.05	− 0.14**	− 0.01	0.13**	0.10*	0.02	− 0.01
(4) Psychological distress				1	− 0.15**	0.15**	0.05	0.14**	0.06	− 0.05	− 0.06	− 0.05	− 0.01
(5) Authoritative					1	− 0.30**	0.20**	− 0.06	0.04	0.07	0.06	− 0.07	− 0.02
(6) Authoritarian						1	0.10*	0.13**	− 0.02	− 0.19**	− 0.18*	0.07	− 0.03
(7) Permissive							1	0.08*	− 0.05	− 0.04	− 0.04	0.06	0.07
(8) Age								1	0.25*	− 0.21**	0.22**	0.04	0.01
(9) Sex									1	0.15*	0.04	− 0.05	− 0.08
(10) Mother's education										1	0.52**	− 0.33**	− 0.04
(11) Father's education											1	− 0.19*	0.06
(12) Mother's job												1	− 0.01
(13) Father's job													1

**Correlation is significant at the 0.01 level

*Correlation is significant at the 0.05 level

### Path analysis results

#### Model 1

**Fig. 1:**
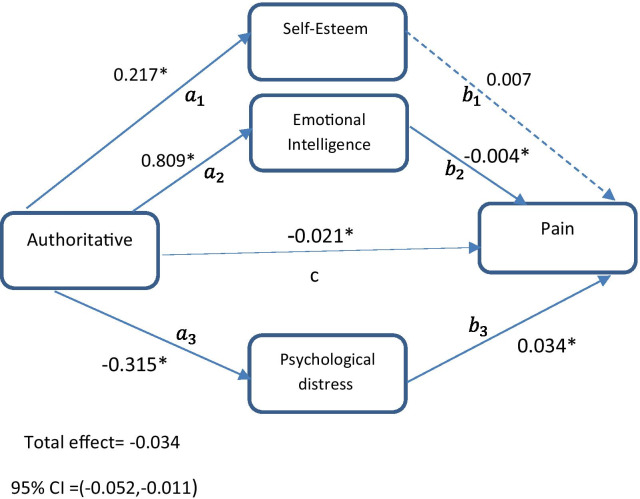
The chart and path coefficients of the mediators in relation to the authoritative parenting style and pain. Age, mother’s education and father’s education were controlled in the model. Non-significant coefficients are shown with dotted lines. *Zero not include in 95% Bayesian credible interval

To evaluate the fitness of the first model, the effect of authoritative style on pain in the presence of three mediators was calculated. Our objective was to evaluate whether the relationship between authoritative parenting style and chronic pain was mediated by self-esteem, emotional intelligence, and psychological distress (Fig. 1). Direct path from authoritative style to pain (c path) was significant (β = − 0.021, 95% CI = − 0.040 to − 0.002). The authoritative style was significantly associated with self-esteem (β = 0.217, 95% CI = 0.153–0.304), emotional intelligence (β = 0.809, 95% CI = 0.344–1.194) and psychological distress (β = − 0.315, 95% CI = − 0.518 to − 0.165) (a paths). The direct effects of emotional intelligence (β = − 0.004, 95% CI = − 0.009 to − 0.001) and psychological distress (β = 0.034, 95% CI = 0.021–0.046) on pain (b paths) were significant, whereas self-esteem (β = 0.007, 95% CI = − 0.021 to 0.032) was not significantly associated with pain (Table 3, Fig. 1). The results in the first model revealed that the indirect path from authoritative style to pain through emotional intelligence (β = − 0.003, 95% CI = − 0.008 to − 0.003) and psychological distress (β = − 0.010, 95% CI = − 0.021 to − 0.004) was significant. The indirect path from authoritative style to pain via self-esteem (β = 0.002, 95% CI = − 0.005 to 0.006) was not significant (Table 3). The total effect of authoritative style on chronic pain (β = − 0.034, 95% CI = − 0.052 to − 0.011) was significant (Fig. 1, Table 3). The first path model was of good fit [PPP = 0.545]. The parameter estimation of the first model is shown in Table 3.

**Table 3:** Results for authoritative parenting style as predictor, three parallel mediators (self-esteem, emotional intelligence, psychological distress) and binary outcome (pain)

Path	Estimate	SD	95% C.I.	
			Lower	Upper
*Direct effect*				
$a_{1}$	0.217*	0.038	0.153	0.304
$a_{2}$	0.809*	0.209	0.344	1.194
$a_{3}$	− 0.315*	0.091	− 0.518	− 0.165
$b_{1}$	0.007	0.012	− 0.021	0.032
$b_{2}$	− 0.004*	0.003	− 0.009	− 0.001
$b_{3}$	0.034*	0.006	0.021	0.046
C	− 0.021*	0.012	− 0.040	− 0.002
*Indirect effect*				
$a_{1} b_{1}$	0.002	0.003	− 0.005	0.006
$a_{2} b_{2}$	− 0.003*	0.002	− 0.008	− 0.003
$a_{3} b_{3}$	− 0.010*	0.004	− 0.021	− 0.004
*Total effect*	− 0.034*	0.012	− 0.052	− 0.011

$a_{1}$ represents the direct path from authoritative style to self-esteem; $a_{2}$ represents the direct path from authoritative style to emotional intelligence; $a_{3}$ represents the direct path from authoritative style to psychological distress; $b_{1}$ represents the direct path from self-esteem to pain; $b_{2}$ represents the direct path from emotional intelligence to pain; $b_{3}$ represents the direct path from psychological distress to pain; c represents the direct path from authoritative style to pain; $a_{1} b_{1}$ represents the indirect path from authoritative style to pain through self-esteem; $a_{2} b_{2}$ represents the indirect path from authoritative style to pain through emotional intelligence; $a_{3} b_{3}$ represents the indirect path from authoritative style to pain through psychological distress; SD, Posterior standard deviation; C.I, 95% Bayesian credible interval

*Zero not include in 95% credible interval. Age, mother’s education and father’s education were controlled in the model

#### Model 2

**Fig. 2:**
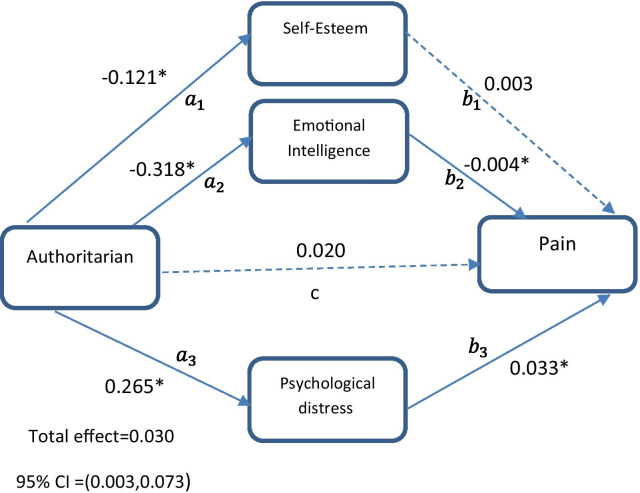
The chart and path coefficients of the mediators in relation to the authoritarian parenting style and pain. Age, mother’s education and father’s education were controlled in the model. Non-significant coefficients are shown with dotted lines. *Zero not include in 95% Bayesian credible interval

The second model was planned to evaluate whether the relationship between authoritarian parenting style and chronic pain was mediated by self-esteem, emotional intelligence, and psychological distress (Fig. 2). The direct effect of authoritarian style on pain (c path) was not significant (β = 0.020, 95% CI = − 0.018 to 0.064). The authoritarian style was significantly associated with self-esteem (β = − 0.121, 95% CI = − 0.184 to − 0.039), emotional intelligence (β = − 0.318, 95% CI = − 0.645 to − 0.076), and psychological distress (β = 0.265, 95% CI = 0.120–0.372) (Fig. 2, Table 4). The direct effects of emotional intelligence (β = − 0.004, 95% CI = − 0.008 to − 0.002) and psychological distress (β = 0.033, 95% CI = 0.022–0.048) on pain were significant, whereas self-esteem (β = 0.003, 95% CI = − 0.015 to 0.027) was not significantly associated with pain (Fig. 2, Table 4). The results in this model revealed that the indirect path from authoritarian style to pain through emotional intelligence (β = 0.001, 95% CI = 0.001–0.003) and psychological distress (β = 0.008, 95% CI = 0.004–0.016) was significant. The indirect path from authoritarian style to pain via self-esteem (β = 0.00, 95% CI = − 0.004 to 0.002) was not significant (Fig. 2, Table 4). The total effect of authoritarian style on chronic pain (β = 0.030, 95% CI = 0.003–0.073) was significant (Fig. 2, Table 4). The second path model was of good fit [PPP = 0.417]. The parameter estimation of the second model is shown in Table 4.

**Table 4:** Results for authoritarian parenting style as predictor, three parallel mediators (self-esteem, emotional intelligence, psychological distress) and binary outcome (pain)

Path	Estimate	SD	95% C.I.	
			Lower	Upper
*Direct effect*				
$a_{1}$	− 0.121*	0.035	− 0.184	− 0.039
$a_{2}$	− 0.318*	0.179	− 0.645	− 0.076
$a_{3}$	0.265*	0.067	0.120	0.372
$b_{1}$	0.003	0.011	− 0.015	0.027
$b_{2}$	− 0.004*	0.002	− 0.008	− 0.002
$b_{3}$	0.033*	0.007	0.022	0.048
C	0.020	0.021	− 0.018	0.064
*Indirect effect*				
$a_{1} b_{1}$	0.000	0.001	− 0.004	0.002
$a_{2} b_{2}$	0.001*	0.001	0.001	0.003
$a_{3} b_{3}$	0.008*	0.003	0.004	0.016
*Total effect*	0.030*	0.021	0.003	0.073

$a_{1}$ represents the direct path from authoritarian style to self-esteem; $a_{2}$ represents the direct path from authoritarian style to emotional intelligence; $a_{3}$ represents the direct path from authoritarian style to psychological distress; $b_{1}$ represents the direct path from self-esteem to pain; $b_{2}$ represents the direct path from emotional intelligence to pain; $b_{3}$ represents the direct path from psychological distress to pain; c represents the direct path from authoritarian style to pain; $a_{1} b_{1}$ represents the indirect path from authoritarian style to pain through self-esteem; $a_{2} b_{2}$ represents the indirect path from authoritarian style to pain through emotional intelligence; $a_{3} b_{3}$ represents the indirect path from authoritarian style to pain through psychological distress; SD, Posterior standard deviation; C.I, 95% Bayesian credible interval

*Zero not include in 95% credible interval. Age, mother’s education and father’s education were controlled in the model.

#### Model 3

**Fig. 3:**
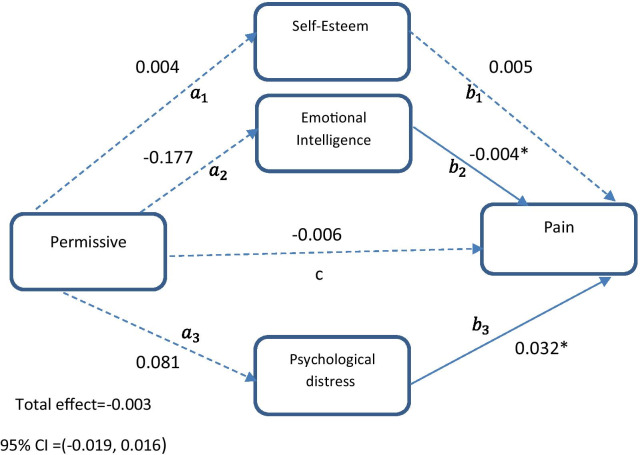
The chart and path coefficients of the mediators in relation to the permissive parenting style and pain. Age, mother’s education and father’s education were controlled in the model. Non-significant coefficients are shown with dotted lines. *Zero not include in 95% Bayesian credible interval

Our objective in model 3 was to identify if the relationship between permissive style and chronic pain was mediated by self-esteem, emotional intelligence, and psychological distress (Fig. 3). Direct path from permissive style to pain (β = − 0.006, 95% CI = − 0.020 to 0.014) was not significant (c path). The direct effects of permissive style on self-esteem (β = 0.004, 95% CI = − 0.078 to 0.081), emotional intelligence (β = − 0.177, 95% CI = − 0.516 to 0.190), and psychological distress (β = 0.081, 95% CI = − 0.077 to 0.276) were not significant (Table 5, Fig. 3). The direct paths from emotional intelligence (β = − 0.004, 95% CI = − 0.010 to − 0.001) and psychological distress (β = 0.032, 95% CI = 0.020–0.045) to pain (b paths) were significant, whereas self-esteem (β = 0.005, 95% CI = − 0.019 to 0.031) was not significantly associated with pain (Table 5, Fig. 3). The results in this model revealed that the indirect paths from permissive style to pain through self-esteem (95% CI = − 0.001 to 0.001), emotional intelligence (95% CI = − 0.001 to 0.003), and psychological distress (95% CI = − 0.003 to 0.009) were not significant. The total effect of permissive style on chronic pain (β = − 0.003, 95% CI = − 0.019 to 0.016) was not significant (Fig. 3, Table 5). The third path model was of good fit [PPP = 0.417]. The parameter estimation of the third model is shown in Table 5.

**Table 5:** Results for permissive parenting style as predictor, three parallel mediators (self-esteem, emotional intelligence, psychological distress) and binary outcome (pain)

Path	Estimate	SD	95% C.I.	
			Lower	Upper
*Direct effect*				
$a_{1}$	0.004	0.039	− 0.078	0.081
$a_{2}$	− 0.177	0.182	− 0.516	0.190
$a_{3}$	0.081	0.086	− 0.077	0.276
$b_{1}$	0.005	0.012	− 0.019	0.031
$b_{2}$	− 0.004*	0.003	− 0.010	− 0.001
$b_{3}$	0.032*	0.006	0.020	0.045
C	− 0.006	0.009	− 0.020	0.014
*Indirect effect*				
$a_{1} b_{1}$	0.000	0.000	− 0.001	0.001
$a_{2} b_{2}$	0.001	0.001	− 0.001	0.003
$a_{3} b_{3}$	0.003	0.003	− 0.003	0.009
*Total effect*	− 0.003	0.009	− 0.019	0.016

$a_{1}$ represents the direct path from permissive style to self-esteem; $a_{2}$ represents the direct path from permissive style to emotional intelligence; $a_{3}$ represents the direct path from permissive style to psychological distress; $b_{1}$ represents the direct path from self-esteem to pain; $b_{2}$ represents the direct path from emotional intelligence to pain; $b_{3}$ represents the direct path from psychological distress to pain; c represents the direct path from permissive style to pain; $a_{1} b_{1}$ represents the indirect path from permissive style to pain through self-esteem; $a_{2} b_{2}$ represents the indirect path from permissive style to pain through emotional intelligence; $a_{3} b_{3}$ represents the indirect path from permissive style to pain through psychological distress; SD, Posterior standard deviation; C.I, 95% Bayesian credible interval

*Zero not include in 95% credible interval. Age, mother’s education and father’s education were controlled in the model.

Totally, our results identified that emotional intelligence and psychological distress significantly mediated the effects of authoritative and authoritarian parenting styles on pain, whereas self-esteem did not.

## Discussion

Chronic pain is a complex and distressing problem which is very common in childhood and adolescence [64]. Recent studies showed that adolescents with chronic pain are at risk of lower educational attainment, lower household income, unemployment, and a number of psychological disorders later in their life [4, 65, 66]. Therefore, investigating the factors which influence chronic pain will be effective in managing this common problem. In the present study, we aimed to investigate the mediating role of self-esteem, emotional intelligence, and psychological distress in the association between parenting styles and chronic pain.

As hypothesized, emotional intelligence and psychological distress significantly mediated the effects of authoritative and authoritarian styles on chronic pain. The present findings are consistent with Palermo and Champers’ model, which posits that adolescents’ emotional symptoms may mediate the relationship between family functioning and pediatric pain [24]. Although there is growing research interest in specific family functioning variables that may influence chronic pain, there are also a number of gaps in the literature regarding our understanding of the effects of parenting styles on adolescent chronic pain [8]. Our study extended previous research by exploring innovative models linking parenting styles to chronic pain in adolescents.

The manners in which parents interact with their children play a crucial role to the emotional development of adolescents. There are different ways in which different parenting styles might influence emotional intelligence in adolescents. Adolescents raised in authoritative households are often encouraged to engage in verbal reasoning exchanges with their parents. They are permitted to express their opinions and minds. Therefore, they have better emotional and communication skills, more ability to access emotion regulation strategies and they can demonstrate more socially adaptive behavior [22]. Authoritarian parenting has inversely adverse effects on adolescents’ emotions. Adolescents raised by authoritarian parents tend to show a limited range of emotions as they are given few opportunities to express their thoughts and feelings at home. Hence, when they experience unfavorable situations, they may “shutdown,” become emotionally withdrawn, or quiet. A very recent study conducted among 1593 students in Vietnam revealed that students who have lack of freedom to have their own decisions, and are overprotected by parents are more likely to have difficulties in understanding their own and others’ feelings and expressing their internal emotions to others, which, in turn, leads to poor relationships with family and friends [30]. Nguyen and colleagues (2020) argued that overprotectiveness and authoritarianism from parents are associated with lower emotional intelligence among children and adolescents, while parents who share healthy and warm relationships with their children develop higher emotional intelligence in them [30].

Emotional intelligence may, in turn, affect the experience of pain in adolescents. Doherty et al. (2017) suggested that emotional intelligence is a useful means of emotional management of pain [67]. It has been proposed that individuals with lower levels of emotional intelligence are more likely to catastrophize about their pain [67]. Catastrophizing, in turn, intensifies the experience of pain [68, 69]. Some other studies discussed negative affectivity, i.e. experiencing negative emotions, as a significant mediator of the relationship between emotional intelligence and the experience of pain [25]. Altogether, the present findings indicate that the manner in which adolescents are raised by their parents—specifically authoritative and authoritarian parenting styles—may influence emotional intelligence in adolescents, which, in turn, can affect the experience of pain by them.

Children and adolescents from authoritative homes have also more ability to regulate their stress. Hence, they have less internalized distress, unlike those from authoritarian ones. Recently, in their valuable review, Gorostiaga and their colleagues (2019) argued that harsh control by parents, which is related to authoritarian style, is positively associated with adolescent anxiety and depression [31]. On the other hand, there are pieces of evidence showing that stress, anxiety, and depression negatively affect the experience of chronic pain in children and adults [37, 70, 71]. Borges Dario and colleagues (2020) in their recent prospective cohort study showed that childhood psychological distress characterized by symptoms of depression, anxiety, and stress increases the risk of the development of spinal pain in adolescents [72]. Also, a 27-year longitudinal study has revealed that higher depressive symptoms at age 16 predicted higher pain intensity in adulthood [73]. Genetic vulnerability, common neurobiological pathways or shared precipitating environmental factors may be possible mechanisms for the relationship between depression and pain [39, 74]. In summary, based on our findings, authoritative and authoritarian parenting styles might both negatively or positively influence chronic pain in adolescents through psychological distress which is characterized by symptoms of depression, stress, and anxiety.

As expected, our data provided no evidence for the direct or indirect association between permissive parenting style and chronic pain. Findings on permissive parenting style are inconclusive. For example, while some studies suggested permissive style as an important predictor of aggressive behavior [75] and internalizing problems among children [76], other studies [77, 78] have found that individuals raised in permissive households are less anxious and depressed compared to those in authoritarian households. More recently, Ada et al. (2018) reported that most adolescents diagnosed with dissociative disorder had mothers with permissive parenting style [79]. However, Argyriou et al. (2016) have found no significant association between permissive parenting and emotional intelligence [22]. To describe these controversies, research has suggested that some cultures may be more tolerant to permissive parenting than others [21]. However, further research in this area is needed.

Our data provided no evidence that the relationship between parenting style and chronic pain is mediated by self-esteem. This finding is in contrast to our hypothesis and appears to contradict our previous report [5] that self-esteem could be an important factor in the development or maintenance of chronic pain in adolescents. In the present study, SEM models were performed to estimate the effects of each independent variable on pain in the presence of three mediating variables. Investigating three mediators in one model allowed the calculation of the specific indirect effect of each mediator, conditional on the inclusion of other mediators in the model. It is quite possible that these variables shared some information regarding their mediating effects on the relationship between parenting styles and chronic pain. However, we cannot and do not present these findings as the final word on this topic, and further research is necessary in this area.

### Limitations

Despite the promising implications of this study, a number of study limitations are worth noting. The data are cross-sectional, and, therefore, our findings do not elucidate a causal relationship. Although many authors use “causal” language in the case of path analysis, causal relationships cannot be achieved through statistical analyses, but only through study designs such as experimental manipulation [80]. Another limitation of this study is related to pain assessment. Assessment of chronic pain based on the self-report questionnaire and not on clinical examination might be influenced by response bias [81]. Moreover, there are certainly other mediators not appraised in this study that also affect the relationship between chronic pain and parenting styles. Further research with prospective design which assesses a wider array of potential mediating factors is needed.

## Conclusion

In sum, maladaptive parenting styles, especially those characterized by high scores in demandingness and low in responsiveness have a negative effect on chronic pain in adolescents whereas authoritative parenting style might reduce pain vulnerability in them. To date, interventions for persons with chronic pain have mostly targeted individual rather than family variables. Little attention has been paid to the role that parent–adolescent communication may play in the development or exacerbation of chronic pain in adolescents. The current results support the notion that interventions targeting effective parent–adolescent communication may be an important part of chronic pain management in adolescents. It is very important for the parents to understand the importance of using effective parenting styles and to gain the knowledge and skills necessary to support their adolescents to fulfill their psychological needs. Moreover, the results provide rationale for targeting emotional intelligence and psychological distress in adolescents by explicitly teaching effective communication skills, expressing opinions and minds, emotion regulation strategies and socially adaptive behaviors. Inclusion of psycho-educational interventions for adolescents and their parents as a part of chronic pain management may be an integral part of best practice to reduce development, maintenance, or exacerbation of chronic pain in adolescents.

## Data Availability

The datasets used and/or analyzed during the current study are available from the corresponding author on reasonable request.

## Abbreviations

CI: Credible interval
DASS: Depression, Anxiety, and Stress Scale
EI: Emotional intelligence
ICD‐11: 11Th Revision of the International Classification of Diseases
MCMC: Markov Chain Monte Carlo
PPP: Posterior Predictive *P*-value
RSES: Rosenberg’s Self-Esteem Scale
SEM: Structural equation modelling
SD: Standard deviation
TEIQue–ASF: Trait Emotional Intelligence Questionnaire–Adolescent Short Form

## References

[1] Gobina I, Villberg J, Välimaa R, Tynjälä J, Whitehead R, Cosma A (2019). Prevalence of self-reported chronic pain among adolescents: evidence from 42 countries and regions. Eur J Pain.

[2] Rosenbloom BN, Rabbitts JA, Palermo TM (2017). A developmental perspective on the impact of chronic pain in late adolescence and early adulthood: implications for assessment and intervention. Pain.

[3] Treede R-D, Rief W, Barke A, Aziz Q, Bennett MI, Benoliel R (2015). A classification of chronic pain for ICD-11. Pain.

[4] Murray CB, Groenewald CB, de la Vega R, Palermo TM (2020). Long-term impact of adolescent chronic pain on young adult educational, vocational, and social outcomes. Pain.

[5] Shaygan M, Karami Z (2020). Chronic pain in adolescents: the predictive role of emotional intelligence, self-esteem and parenting style. Int J Community Based Nurs Midwifery.

[6] Bevers K, Watts L, Kishino ND, Gatchel RJ (2016). The biopsychosocial model of the assessment, prevention, and treatment of chronic pain. US Neurol.

[7] Cousins LA, Kalapurakkel S, Cohen LL, Simons LE (2015). Topical review: resilience resources and mechanisms in pediatric chronic pain. J Pediatr Psychol.

[8] Palermo TM, Valrie CR, Karlson CW (2014). Family and parent influences on pediatric chronic pain: a developmental perspective. Am Psychol.

[9] Guite JW, Russell BS, Homan KJ, Tepe RM, Williams SE (2018). Parenting in the context of children’s chronic pain: balancing care and burden. Children.

[10] Chow ET, Otis JD, Simons LE (2016). The longitudinal impact of parent distress and behavior on functional outcomes among youth with chronic pain. J Pain.

[11] Anno K, Shibata M, Ninomiya T, Iwaki R, Kawata H, Sawamoto R (2015). Paternal and maternal bonding styles in childhood are associated with the prevalence of chronic pain in a general adult population: the Hisayama Study. BMC Psychiatry.

[12] Shibata M, Ninomiya T, Anno K, Kawata H, Iwaki R, Sawamoto R (2016). Perceived inadequate care and excessive overprotection during childhood are associated with greater risk of sleep disturbance in adulthood: the Hisayama Study. BMC Psychiatry.

[13] Yoshida T, Taga C, Matsumoto Y, Fukui K (2005). Paternal overprotection in obsessive-compulsive disorder and depression with obsessive traits. Psychiatry Clin Neurosci.

[14] Agostini A, Rizzello F, Ravegnani G, Gionchetti P, Tambasco R, Ercolani M (2010). Parental bonding and inflammatory bowel disease. Psychosomatics.

[15] Shibata M, Ninomiya T, Anno K, Kawata H, Iwaki R, Sawamoto R (2020). Parenting style during childhood is associated with the development of chronic pain and a patient's need for psychosomatic treatment in adulthood: a case–control study. Medicine..

[16] Maccoby EE, Martin JA, Mussen PH, Hetherington EM (1983). Socialization in the context of the family: parent–child interaction. Handbook of child psychology: vol 4: socialization, personality and social development; E Mavis Hetherington, volume editor.

[17] Baumrind D (1971). Current patterns of parental authority. Dev Psychol.

[18] Baumrind D, Damon T (1989). Rearing competent children. Child development today and tomorrow.

[19] Grolnick WS (2012). The relations among parental power assertion, control, and structure. Hum Dev.

[20] Kandemir G, Hesapcioglu ST, Kurt ANC (2018). What are the psychosocial factors associated with migraine in the child? Comorbid psychiatric disorders, family functioning, parenting style, or mom’s psychiatric symptoms?. J Child Neurol.

[21] Pinquart M, Kauser R (2018). Do the associations of parenting styles with behavior problems and academic achievement vary by culture? results from a meta-analysis. Cult Divers Ethnic Minor Psychol.

[22] Argyriou E, Bakoyannis G, Tantaros S (2016). Parenting styles and trait emotional intelligence in adolescence. Scand J Psychol.

[23] Shafipour SZ, Sheikhi A, Mirzaei M, KazemnezhadLeili E (2015). Parenting styles and its relation with children behavioral problems. J Holist Nurs Midwifery.

[24] Palermo TM, Chambers CT (2005). Parent and family factors in pediatric chronic pain and disability: an integrative approach. Pain.

[25] Ruiz-Aranda D, Salguero JM, Fernández-Berrocal P (2011). Emotional intelligence and acute pain: the mediating effect of negative affect. J Pain.

[26] Petrides K, Sangareau Y, Furnham A, Frederickson N (2006). Trait emotional intelligence and children's peer relations at school. Soc Dev.

[27] Baudry AS, Grynberg D, Dassonneville C, Lelorain S, Christophe V (2018). Sub-dimensions of trait emotional intelligence and health: a critical and systematic review of the literature. Scand J Psychol.

[28] Matthews G, Zeidner M, Roberts RD, Cooper CL, Quick JC (2017). Emotional intelligence, health, and stress. The handbook of stress and health: a guide to research and practice.

[29] Zeidner M, Matthews G, Roberts RD (2012). The emotional intelligence, health, and well-being nexus: What have we learned and what have we missed?. Appl Psychol Health Well Being.

[30] Nguyen Q-AN, Tran T, Tran T-A, Nguyen TA, Fisher J (2020). Perceived parenting styles and emotional intelligence among adolescents in Vietnam. Fam J.

[31] Gorostiaga A, Aliri J, Balluerka N, Lameirinhas J (2019). Parenting styles and internalizing symptoms in adolescence: a systematic literature review. Int J Environ Res Public Health.

[32] Baumrind D (1991). The influence of parenting style on adolescent competence and substance use. J Early Adolesc.

[33] Liu Y, Merritt DH (2018). Examining the association between parenting and childhood depression among Chinese children and adolescents: a systematic literature review. Child Youth Serv Rev.

[34] Lumley MA, Cohen JL, Borszcz GS, Cano A, Radcliffe AM, Porter LS (2011). Pain and emotion: a biopsychosocial review of recent research. J Clin Psychol.

[35] Soltani S, Kopala-Sibley DC, Noel M (2019). The co-occurrence of pediatric chronic pain and depression. Clin J Pain.

[36] Angst F, Benz T, Lehmann S, Wagner S, Simmen BR, Sandòr PS (2020). Extended overview of the longitudinal pain-depression association: a comparison of six cohorts treated for specific chronic pain conditions. J Affect Disord.

[37] Shaygan M (2017). Intensity of depression, its predictive and mediating factors in the patients with chronic headache. J Kurdistan Univ Med Sci.

[38] Koechlin H, Coakley R, Schechter N, Werner C, Kossowsky J (2018). The role of emotion regulation in chronic pain: a systematic literature review. J Psychosom Res.

[39] Sheng J, Liu S, Wang Y, Cui R, Zhang X (2017). The link between depression and chronic pain: neural mechanisms in the brain. Neural Plast.

[40] Goesling J, Clauw DJ, Hassett AL (2013). Pain and depression: an integrative review of neurobiological and psychological factors. Curr Psychiatry Rep.

[41] Horenstein A, Potter CM, Heimberg RG (2018). How does anxiety sensitivity increase risk of chronic medical conditions?. Clin Psychol Sci Pract.

[42] Rosenberg M, Schooler C, Schoenbach C (1989). Self-esteem and adolescent problems: modeling reciprocal effects. Am Sociol Rev.

[43] Ross AC, Simons LE, Feinstein AB, Yoon IA, Bhandari RP (2018). Social risk and resilience factors in adolescent chronic pain: examining the role of parents and peers. J Pediatr Psychol.

[44] Jadon PS, Tripathi S (2017). Effect of authoritarian parenting style on self esteem of the child: a systematic review. Int J Adv Res Innov Ideas Educ.

[45] Petrides KV, Parker JDA, Saklofske DH, Stough C (2009). Psychometric properties of the Trait Emotional Intelligence Questionnaire (TEIQue). Assessing emotional intelligence.

[46] Ashouri A, Jamil L, Alimoradi H, Aghedi M (2020). Psychometric properties of Farsi version of Trait Emotional Intelligence Questionnaire-Adolescent Short Form. Iran J Psychiatry Behav Sci..

[47] Rajabi G, Bohlol N (2007). Assessing the reliability and validity of Rosenberg self-esteem in the first year of the University of Shahid Chamran. Educ Psychol Res.

[48] Shapurian R, Hojat M, Nayerahmadi H (1987). Psychometric characteristics and dimensionality of a Persian version of Rosenberg Self-esteem Scale. Percept Mot Skills.

[49] Lovibond PF, Lovibond SH (1995). The structure of negative emotional states: comparison of the Depression Anxiety Stress Scales (DASS) with the Beck Depression and Anxiety Inventories. Behav Res Ther.

[50] Asghari A, Saed F, Dibajnia P (2008). Psychometric properties of the Depression Anxiety Stress Scales-21 (DASS-21) in a non-clinical Iranian sample. Int J Psychol.

[51] Farahini F, Afrooz GHA, Rasoolzadeh-Tabatabaiee K (2014). The relationship between parenting styles, shyness and creativity in the gifted. J Sch Psychol.

[52] Minaei A, Nikzad S (2017). The factor structure and validity of the Persian version of the Baumrind parenting style inventory. J Fam Res.

[53] Field A (2013). Discovering statistics using IBM SPSS statistics.

[54] Kim H-Y (2013). Statistical notes for clinical researchers: assessing normal distribution (2) using skewness and kurtosis. Restor Dent Endod.

[55] Kline RB (2015). Principles and practice of structural equation modeling.

[56] Tabachnick B, Fidell L (2013). Using multivariate statistics.

[57] Schafer JL (1997). Analysis of incomplete multivariate data.

[58] Muthén LK, Muthén BO. Mplus user’s guide. 6th ed. Los Angeles: Muthén & Muthén; 1998–2010.

[59] Yuan Y, MacKinnon DP (2009). Bayesian mediation analysis. Psychol Methods.

[60] Muthén B. Bayesian analysis in Mplus: a brief introduction. Citeseer; 2010.

[61] Miočević M, Gonzalez O, Valente MJ, MacKinnon DP (2018). A tutorial in Bayesian potential outcomes mediation analysis. Struct Equ Modeling.

[62] Asparouhov T, Muthén B. Bayesian analysis using Mplus: technical implementation. Citeseer; 2010.

[63] Muthén B, Asparouhov T (2012). Bayesian structural equation modeling: a more flexible representation of substantive theory. Psychol Methods.

[64] Fayaz A, Croft P, Langford R, Donaldson L, Jones G (2016). Prevalence of chronic pain in the UK: a systematic review and meta-analysis of population studies. BMJ Open.

[65] Friedrichsdorf SJ, Giordano J, Desai Dakoji K, Warmuth A, Daughtry C, Schulz CA (2016). Chronic pain in children and adolescents: diagnosis and treatment of primary pain disorders in head, abdomen, muscles and joints. Children.

[66] Johannes CB, Le TK, Zhou X, Johnston JA, Dworkin RH (2010). The prevalence of chronic pain in United States adults: results of an Internet-based survey. J Pain.

[67] Doherty EM, Walsh R, Andrews L, McPherson S (2017). Measuring emotional intelligence enhances the psychological evaluation of chronic pain. J Clin Psychol Med Settings.

[68] Shaygan M, Shayegan L (2019). Understanding the relationship between spiritual well-being and depression in chronic pain patients: the mediating role of pain catastrophizing. Pain Manag Nurs.

[69] Beltran-Alacreu H, López-de-Uralde-Villanueva I, Calvo-Lobo C, Fernández-Carnero J, La Touche R (2018). Clinical features of patients with chronic non-specific neck pain per disability level: a novel observational study. Rev Assoc Med Bras.

[70] Shaygan M, Yazdanpanah M (2020). Prevalence and predicting factors of chronic pain among workers of petrochemical and petroleum refinery plants. Int J Occup Environ Med.

[71] Huguet A, Tougas ME, Hayden J, McGrath PJ, Stinson JN, Chambers CT (2016). Systematic review with meta-analysis of childhood and adolescent risk and prognostic factors for musculoskeletal pain. Pain.

[72] Borges Dario A, Kamper SJ, Williams C, Straker L, O’Sullivan P, Schütze R (2020). Psychological distress in early childhood and the risk of adolescent spinal pain with impact: a longitudinal cohort study. SSRN.

[73] Leino-Arjas P, Rajaleid K, Mekuria G, Nummi T, Virtanen P, Hammarström A (2018). Trajectories of musculoskeletal pain from adolescence to middle age: the role of early depressive symptoms, a 27-year follow-up of the Northern Swedish Cohort. Pain.

[74] Hooten WM (2016). Chronic pain and mental health disorders: shared neural mechanisms, epidemiology, and treatment. Mayo Clin Proc.

[75] Llorca A, Cristina Richaud M, Malonda E (2017). Parenting, peer relationships, academic self-efficacy, and academic achievement: direct and mediating effects. Front Psychol.

[76] Williams LR, Degnan KA, Perez-Edgar KE, Henderson HA, Rubin KH, Pine DS (2009). Impact of behavioral inhibition and parenting style on internalizing and externalizing problems from early childhood through adolescence. J Abnorm Child Psychol.

[77] Chatterjee S (2016). Children’s perspective on parenting styles: a developmental approach. Int J Indian Psychol.

[78] Bakhla AK, Sinha P, Sharan R, Binay Y, Verma V, Chaudhury S (2013). Anxiety in school students: role of parenting and gender. Ind Psychiatry J.

[79] Ada K, Mahour P, Kar S, Agarwal V, Arya A (2018). Study of parenting styles and attachment in adolescents with dissociative disorder. J Indian Assoc Child Adolesc Ment Health.

[80] Barbeau K, Boileau K, Sarr F, Smith K (2019). Path analysis in Mplus: a tutorial using a conceptual model of psychological and behavioral antecedents of bulimic symptoms in young adults. Quant Methods Psychol.

[81] Shaygan M, Böger A, Kröner-Herwig B (2014). Neuropathic sensory symptoms: association with pain and psychological factors. Neuropsychiatr Dis Treat.

